# Effects of holding soft objects during Cyberball tasks under frequent positive feedback

**DOI:** 10.1007/s00221-020-06000-9

**Published:** 2021-01-02

**Authors:** Toshiki Ikeda, Yuji Takeda

**Affiliations:** 1 Graduate School of Comprehensive Human Sciences, University of Tsukuba, Tsukuba, Japan; 2 Human-Centered Mobility Research Center, National Institute of Advanced Industrial Science and Technology (AIST), AIST Tsukuba Central 6, 1-1-1 Higashi, Tsukuba, Ibaraki 305-8566 Japan

**Keywords:** Soft objects, expectation, Cyberball task, Social over-inclusion, contingent negative variation (CNV)

## Abstract

A previous study suggested that holding soft objects enhanced expectations of uncertain events and increased social pain under frequent negative feedback; i.e., higher expectations might have induced more disappointment. The present study examined the effects of holding a soft cushion under frequent positive feedback. Participants (*n* = 42) performed fair-play and over-inclusion blocks in the Cyberball task. Amplitudes of the contingent negative variation (CNV) of event-related brain potentials and subjective ratings of social pain were measured to estimate participants’ expectations and emotions, respectively. CNV amplitudes were higher in the over-inclusion block when participants held the soft than the hard cushion. There was a statistically marginal trend (*p* = .095) for lower social pain scores in the soft cushion condition than the hard cushion condition in contrast to previous findings. These results suggest that holding a soft object does not directly modulate emotions but instead acts through the mediation of enhanced expectations.

## Introduction

It is known that a soft touch can provide feelings of safety and security and can influence attitudes and emotions. Previous experimental studies have reported that touching/holding soft objects has effects on attitudes and emotions in social situations, such as a decrease in negative ratings of other people (Ackerman et al. 2010) and an increase in tolerance for social uncertainty (Van Horen and Mussweiler 2014). Because these studies used a paradigm in which others did not give participants affective feedback, it was unclear whether the effect of touching/holding soft objects could increase positive attitudes or emotions even after the occurrence of negative feedback. Recently, we demonstrated that holding a soft object increased expectations about uncertain forthcoming events and also increased disappointment when negative feedback was given by others (Ikeda and Takeda 2019). The participants in the previous study performed a social interaction task in which we experimentally controlled the degree of social inclusion or exclusion (i.e., a Cyberball task). The expectations about being included with others and social pain induced by exclusion were estimated by electrophysiological and subjective rating measures, respectively. The results indicated higher expectations of being included with others (i.e., a positive emotion before social feedback) and social pain (i.e., a negative emotion after negative feedback) when participants held a soft object compared to a hard object. These results could be explained that participants expected to be included by others if they were holding a soft object, but they were more disappointed if they received negative feedback by being excluded. Therefore, our previous study’s results imply that touching or holding soft objects do not directly modulate attitudes and emotions about uncertain forthcoming events; instead, touching or holding soft objects influences attitudes and/or emotions by enhancing positive expectations about uncertain situations. In the current study, we hypothesize that holding soft objects does not directly modulate emotions but instead acts by enhancing expectations. We hypothesized that if participants are given frequent positive feedback, the enhancement of expectations might decrease disappointments and increase positive emotions.

To examine this hypothesis, following our previous study, we used a Cyberball task, which is a virtual ball-tossing game that experimentally controls the degree of social inclusion and exclusion (Williams et al. 2000; Williams and Jarvis 2006). In the Cyberball task, a participant catches and throws a ball with two other players on a screen. When the participant catches the ball, she/he is required to throw the ball to one of the other two players. Many studies have reported that the subjective ratings of social pain increase if participants are frequently excluded; i.e., the ball is tossed between the other two players on most trials. (Hartgerink et al. 2015), whereas those ratings decrease if participants are frequently included; i.e., over-inclusion, others frequently toss the ball to the participants (Van Beest and Williams 2006; Kawamoto et al. 2012; Niedeggen et al. 2014). In the present Cyberball task, participants performed two types of blocks: *a fair-play* block and *an over-inclusion* block. In the fair-play block, the ball was thrown to the participant and the other two players with equal probability. By contrast, in the over-inclusion block, the ball was thrown with an extremely high probability to the participant, relative to the other players' probabilities. It has been reported that subjective ratings of negative emotions decreased in the over-inclusion block compared to the fair-play block (e.g., Niedeggen et al. 2014; Williams et al. 2000) because the over-inclusion block provides frequent positive feedback in line with expectations. This study's hypothesis predicted that holding soft objects decreases disappointment (i.e., increases positive emotions) by enhancing expectations. This hypothesis contrasts with our previous study, in which holding a soft object increased social pain if the participants were frequently excluded (Ikeda and Takeda, 2019).

The contingent negative variation (CNV) of event-related brain potentials (ERPs) is a slow wave occurring in the interval between the presentation of a warning stimulus and an imperative stimulus requiring a motor response (Walter et al. 1964). Several studies have proposed that expectations and anticipations of an imperative stimulus are related to the CNV amplitude (Van Boxtel and Brunia 1994a, b; Ruchkin et al. 1986). Similar to our previous study (Ikeda and Takeda 2019), we assessed the CNV amplitudes and subjective ratings of social pain to estimate participants’ expectations and emotions, respectively. We also evaluated the P3, which develops after ball movements. It has been reported that the P3 amplitude varies with the subjective probability of ball possession in the Cyberball task. For example, Weschke and Niedeggen (2015) demonstrated that the P3 amplitude was larger when the ball was tossed to participants who had been frequently excluded (violation of subjective probability of ball possession; i.e., a rare event). The CNV develops before the onset of ball movement, whereas the P3 develops after ball movement. Therefore, it is reasonable to consider that the CNV reflects the expectation and the anticipation of the forthcoming stimulus, and the P3 reflects the evaluations of the presented stimulus (Ikeda and Takeda 2019). Subjective social pain ratings were assessed using four statements designed to identify the participants’ subjective experience of self-esteem, belongingness, meaningfulness, and control (Williams et al. 2000). If holding soft objects enhanced expectations and decreased negative emotions, then CNV amplitudes would be larger in the over-inclusion block when participants held a soft object than a hard object. Furthermore, subjective ratings of social pain in the over-inclusion block might decrease when participants held a soft object than a hard object. The probability of inclusion with others was higher than exclusion in this study, and the P3 reflects the subjective probability of ball possession. Therefore, we expected the P3 amplitude to be larger when the ball was tossed between the other two players, which was a rare event.

## Materials and methods

### Participants

Forty-six healthy participants took part in the experiment. The number of participants was predetermined based on past research (Niedeggen et al. 2014), in which data of 40 participants were analyzed to examine the effect of over-inclusion on ERPs. Four participants were excluded from the analyses due to many EEG artifacts (2 participants) or incorrect performance of the task (2 participants). Thus, the data of 42 participants (17 female, *M*_age_ = 22.52, SD_age_ = 2.84; All right-handed except one participant) were analyzed. All participants had a normal or corrected-to-normal vision. They received payment (1250 yen / 1 h) at the end of the experiment. This experiment was approved by the National Institute of Advanced Industrial Science and Technology (AIST) Safety and Ethics committee and was only conducted after each of the participants had given written informed consent.

### Apparatus

The visual stimuli were presented on a 17-inch cathode ray tube display (Sony, Trinitron Multiscan G220) with a resolution of 1280 × 1024 pixels, which was controlled by Windows 7, MATLAB (MathWorks Inc.), and Psychophysics Toolbox (Brainard 1997; Kleiner et al. 2007; Pelli 1997). The refresh rate of the display was set to 60 Hz. The viewing distance was approximately 70 cm.

Two visually similar cushions, one soft and the other hard, were used as the objects to be held. Both cushions were covered with white cotton and were 40 × 40 × 10 cm in size. The soft cushion was made of polyester and weighed about 850 g. The hard cushion was made of polyethylene pipes and weighed about 1000 g. We instructed participants to hold each cushion with their left arms and place it on their thighs.

### Stimuli and procedure

Following our previous study (Ikeda and Takeda 2019), two computer-generated opponents (black outlined squares, 2.1° × 2.1° of visual angle) appeared at the top left and top right locations of the screen, separated by 6.5° of visual angle. Male and female facial photographs representing co-players were depicted on the screen. The facial photographs were emotionally neutral and selected from a facial expression database (Fujimura and Umemura 2018). A player, controlled by the participant (a black outlined square), appeared at the bottom of the screen. A white filled circle represented the ball (diameter 0.82° of visual angle). To precisely inform participants of the ball movements' timing, 1500 ms before the ball began moving, it flickered for 300 ms (disappearing for 50 ms and appearing for 50 ms, three times). The ball visibly traveled over a distance of 3.25° for 1000 ms until another player received it. If participants received the ball, they were required to press a left or right arrow key on a keyboard with their right index or middle finger to toss the ball to the left or right computer-generated player, respectively. Participants were instructed to toss the ball to the left and right players with approximately equal probability. If the computer players received the ball, they held the ball for a random period lasting between 1000 and 2000 ms, and then they tossed the ball to one of the other players. At the beginning of the experiment, we gave participants a cover story indicating that tossing performance was unimportant because the task was to examine mental visualization skills. We instructed participants that black squares (outlined) indicated players; the lower square corresponded to the participant, and others corresponded to computer players. Participants were aware that the other players did not actually exist (i.e., they were computer-generated players). Note that apparatus, stimuli, and the task were identical to the previous study by Ikeda and Takeda (2019) except that facial photographs were presented on the display, and the size of the ball was small.

Each participant performed 8 blocks. Participants held one type of cushion during the first 4 blocks and the other cushion type during the remaining 4 blocks. The order of soft and hard cushion conditions was randomized among participants (20 participants held a soft cushion first). Participants started the experiment after practicing ten trials of the task without holding a cushion. Each cushion condition consisted of two fair-play blocks and two over-inclusion blocks. In the fair-play block, participants received the ball 20 times (inclusion trials) and then tossed it to one of the computer-generated opponents 20 times. In this condition, the computer-generated opponents also tossed the ball to each other 20 times (micro-rejection trials). Thus, each fair-play block consisted of 60 trials. In the over-inclusion block, the participant received and tossed the ball 28 times (over-inclusion trials), and the computer-generated opponents tossed the ball to each other 4 times. Thus, each over-inclusion block also consisted of 60 trials. At the beginning of each cushion condition, participants performed the fair-play block. In the remaining three blocks, they performed one fair-play block and two over-inclusion blocks in a random order. The fair-play block provides baseline experiences of the Cyberball task. Therefore, this manipulation was expected to result in an unbiased mental set for the task at the beginning of each cushion condition. Participants had a short break after each block.

Following the previous studies (Ikeda and Takeda 2019; Kawamoto et al. 2013), during the short breaks between the blocks, participants were required to provide subjective ratings of their social pain by responding to each of the following questions: “I felt liked,” “I felt rejected,” "I felt invisible,” and “I felt powerful” (in Japanese). These questions corresponded to evaluations of self-esteem, belongingness, meaningfulness, and control, respectively, and were rated on a scale ranging from 1 (*not at all*) to 9 (*very much*). Two questions, “I felt liked” and “I felt powerful,” were reverse-scored, such that higher scores for each question indicated a greater level of social pain. We averaged these four questions as the subjective rating of social pain scores. Debriefing followed the end of the eighth block. The total experiment time was about 50 min.

### EEG recordings

The electroencephalographic (EEG) signals were acquired with a digital amplifier (Brain Products, BrainAmp standard system). The silver-silver chloride electrodes were placed at 27 scalp sites: Fp1, Fp2, F7, F3, Fz, F4, F8, FCz, T3, C3, Cz, C4, T4, CPz, T5, P3, Pz, P4, T6, PO7, PO3, POz, PO4, PO8, O1, Oz, and O2, according to the extended international 10–20 system, with AFz as the ground electrode. The EEGs were re-referenced to mathematically averaged earlobes (A1–A2) offline. To monitor blinks and eye movements, vertical and horizontal electrooculograms (EOGs) were also acquired using electrodes placed above and below the right eye and the outer left and right canthi, respectively. The impedance of all electrodes was kept below 10 kΩ. The EEGs and EOGs were digitized at a sampling rate of 1000 Hz and the time constant was set at 10 s. All EEG and EOG signals were low-pass-filtered at 30 Hz with a second-order Butterworth filter to compute the CNV, whereas the 0.1–30 Hz bandpass filter was adopted to compute the P3.

### Data analysis

The time epochs were set between − 2000 ms and 1000 ms and between − 200 ms and 600 ms relative to the onset of ball movement (i.e., the ball’s appearance at the intermediate position between players) to compute CNV and P3, respectively. We examined the epochs in which the computer-generated players threw the ball, after excluding epochs in which the participants threw the ball. We adopted an independent component analysis using EEGLAB version 14.1.1b (Delorme and Makeig 2004) to remove eye-blink-related components. We excluded epochs in which signal changes exceeded ± 50 μV on any of the EEGs from the analysis. We averaged 69.0 and 68.7 CNV epochs in the soft and hard cushion conditions of the fair-play block. We also averaged 52.2 and 53.7 CNV epochs of the soft and hard cushion conditions. Moreover, in the inclusion trials (i.e., the ball tossed to participants in the fair-play block), we averaged 39.57 and 39.45 P3 epochs of the soft and hard cushion conditions. Furthermore, in the micro-rejection trials (i.e., the ball tossed between others in the fair-play block), we averaged 38.86 epochs and 38.57 epochs of the soft and hard cushion conditions. Finally, in the over-inclusion trials (i.e., the ball tossed to the participants in the over-inclusion block), we averaged 54.19 epochs and 53.88 epochs of the soft and hard cushion conditions.

The amplitudes of ERPs were evaluated relative to baseline (the mean amplitude of the − 2000 ms to − 1500 ms window in the CNV; the mean amplitude of the − 200 ms to 0 ms in the P3). In each condition, the CNV was estimated by the mean amplitude at the centroparietal site (average of FCz, Cz, CPz and Pz) between -1000 and 0 ms. The P3 was estimated by the mean amplitude at the centrooccipital site (average of Cz, CPz, Pz and POz) between 320 and 400 ms. These time windows and electrodes were determined from visual inspection of the grand mean waveforms and topographical maps.

A 2-way repeated measure analyses of variance (ANOVA) with cushion condition (soft vs. hard) and block (fair-play vs. over-inclusion) were conducted on subjective ratings of social pain and CNV amplitudes. Also, a 2-way repeated measure ANOVA with cushion (soft vs. hard) and trials (inclusion vs. micro-rejection vs. over-inclusion) was performed to assess the P3. Three-way mixed ANOVAs with cushion (soft vs. hard), block (fair-play vs. over-inclusion), and order was conducted when there were significant effects and/or cushion condition interactions, to rule out possible contamination by effects of cushion condition order (first holding a soft cushion vs. first holding a hard cushion). The significance level was set at 5%.

### Results

### Subjective ratings of social pain

**Fig. 1:**
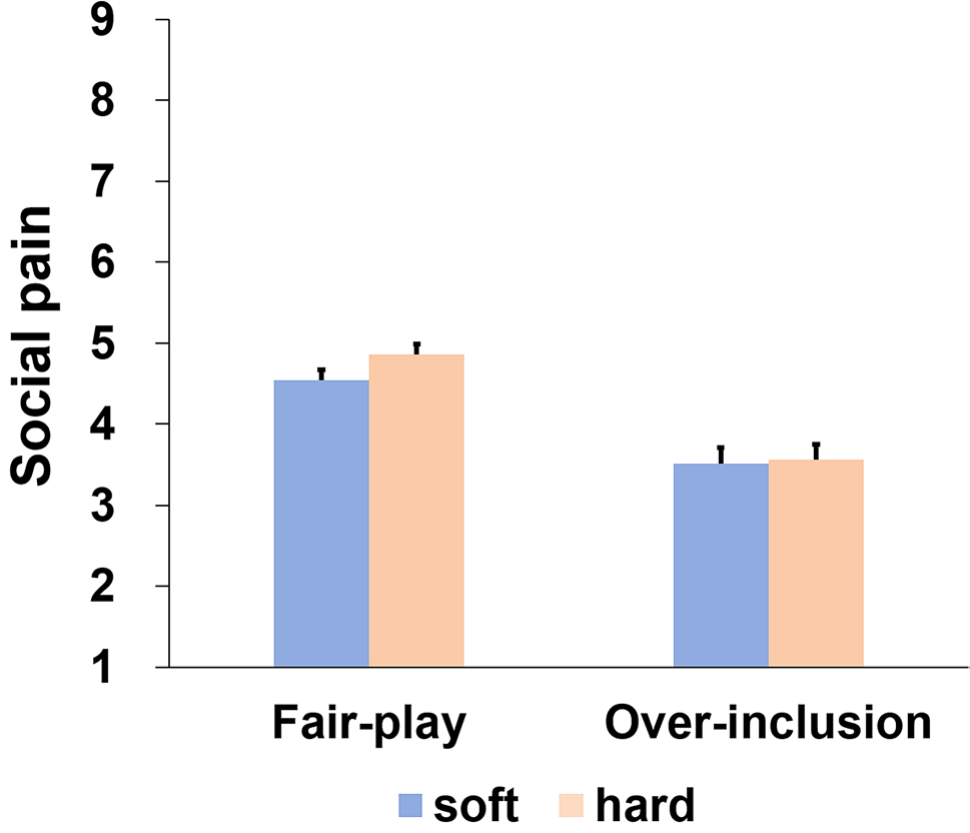
Subjective ratings of social pain (the mean of the four items). Higher scores indicate greater pain. Error bars indicate one standard error of the mean

Figure 1 depicts subjective ratings of social pain in each condition. A 2-way ANOVA was conducted on subjective ratings of social pain to test whether holding a soft compared a hard cushion reduced social pain scores. Results indicated significant main effects of the block suggesting significantly greater social pain in fair-play than over-inclusion blocks (*F* (1, 41) = 43.83, *p* < 0.001, *η*_p_^2^ = 0.52). Moreover, the main effect of the cushion was marginally significant (*F* (1, 41) = 2.92, *p* = 0.095, *η*_p_^2^ = 0.07), indicating that social pain scores in the soft cushion condition were less than in the hard cushion condition. There was no significant interaction between the cushion and social pain conditions (*p* = 0.11).

### CNV

**Fig. 2:**
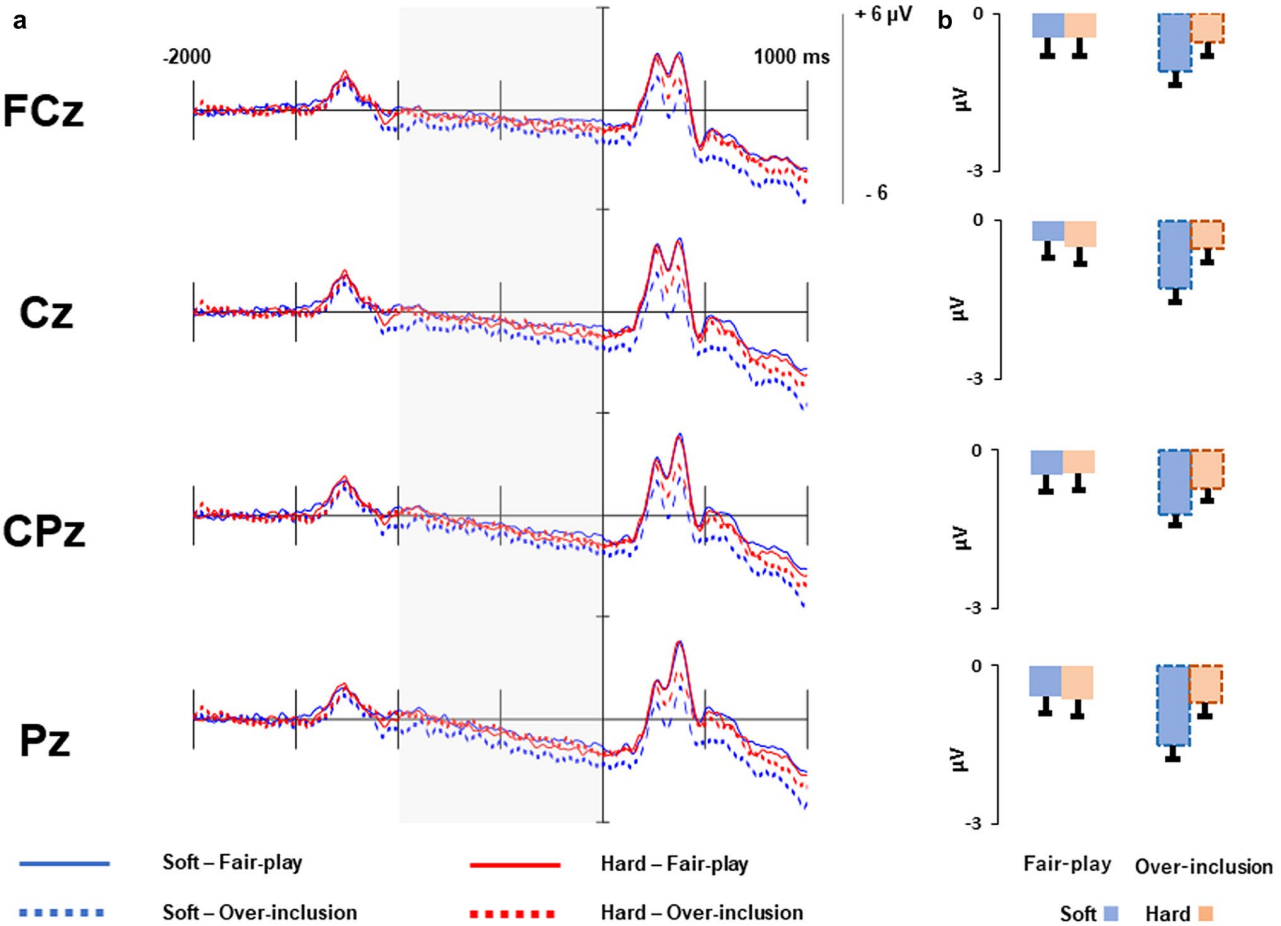
**a** Grand-averaged CNVs at midline for the cushion × block conditions. **b** Mean amplitudes of CNVs in each condition. Error bars indicate one standard error of the mean

Figure 2 shows grand-averaged CNVs time-locked to ball movements, and mean CNV amplitudes. A 2-way ANOVA was conducted on the CNV amplitudes to test whether holding a soft cushion increased expectations more than a hard cushion. Results indicated a significant cushion × block interaction (*F* (1, 41) = 8.93, *p* < 0.01, *η*_p_^2^ = 0.18) for CNV amplitudes. Post-hoc analyses with the Ryan method indicated that CNV amplitudes were significantly larger (more negative) in the soft than the hard cushion condition for the over-inclusion block (*M* = − 1.27 µV vs. *M* = − 0.65 µV, *p* < 0.01), but not for the fair-play block (*M* = − 0.49 µV vs. *M* = − 0.72 µV, *p* = 0.30). Furthermore, the CNV amplitude was significantly larger (more negative) for the over-inclusion block than for the fair-play block in the soft cushion (*M* = − 1.27 µV vs. − 0.49 µV, *p* < 0.001), but not in the hard cushion condition (*M* = − 0.65 µV vs. − 0.72 µV, *p* = 0.76). A significant main effect of block was also observed (*F* (1, 41) = 4.82, *p* < 0.05, *η*_p_^2^ = 0.11), indicating that CNV amplitude was significantly larger (more negative) in the over-inclusion block than in the fair-play block. However, the main effect of cushion was not significant (*p* = 0.23).

Three-way ANOVAs were conducted on the CNV amplitude to examine the effects of the cushion holding order. A cushion (soft vs. hard) × block (fair-play vs. over-inclusion) × order (first holding a soft cushion vs. first holding a hard cushion) ANOVA confirmed the significant cushion × block interaction and the main effect of the block observed in the 2-way ANOVA. Notably, there were no other interactions (*p*s > 0.58) or main effects (*p*s > 0.23), which indicated that the order of holding the cushions had no effect on the CNV amplitude.

### P3

**Fig. 3:**
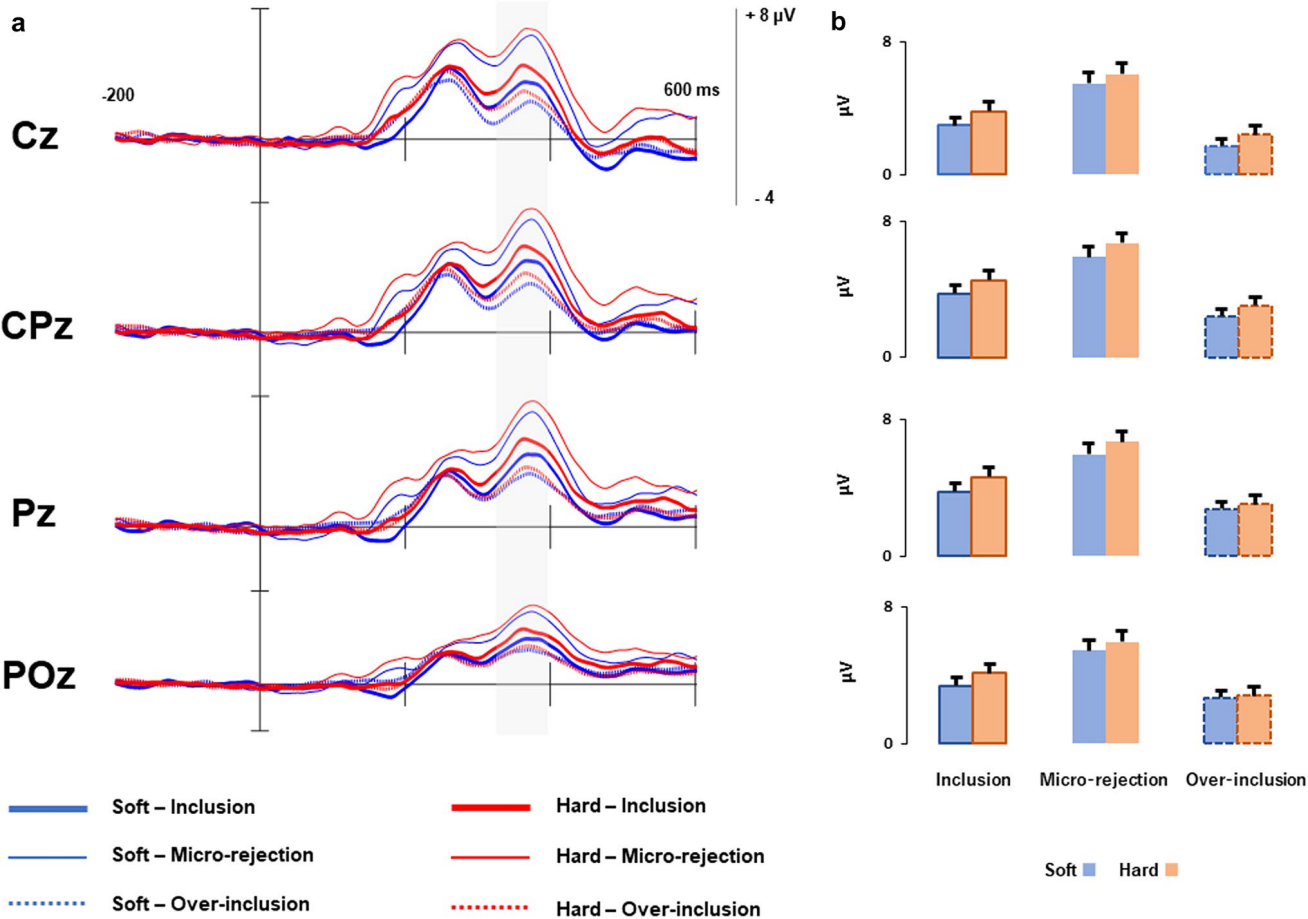
**a** Grand-averaged P3s at midline for the cushion × block conditions. **b** Mean amplitudes of P3s in each condition. Error bars indicate one standard error of the mean

Figure 3 shows grand-averaged P3s time-locked to ball movements and mean P3 amplitudes. A cushion (soft vs. hard) × trials (inclusion vs. micro-rejection vs. over-inclusion) ANOVA was conducted on the P3 amplitudes to examine the processes of evaluating the presented stimulus. Results indicated a significant main effect of trial (*F* (2, 82) = 31.80, *p* < 0.001, *η*_p_^2^ = 0.44). Post-hoc analyses with the Ryan method indicated that the P3 amplitude was significantly larger (more positive) in inclusion trials than in over-inclusion trials; in micro-rejection trials than in over-inclusion trials, and in micro-rejection trials than in inclusion trials (*p*s < 0.001). However, there was neither a main effect of cushion (*F* (1. 41) = 2.12, *p* = 0.15, *η*_p_^2^ = 0.05) nor a significant cushion × trials interaction (*F* (2. 82) = 0.25, *p* = 0.78, *η*_p_^2^ = 0.01).

## Discussion

A previous study suggested that holding soft objects enhanced expectations about uncertain events and increased social pain under frequent negative feedback. To explain this, we hypothesized that holding a soft object does not directly modulate emotions, but instead acts through the mediation of enhanced expectations of being included by others, with higher expectations resulting in higher disappointment when the feedback is negative. If this were the case, then enhanced expectations from holding a soft object would decrease disappointment (i.e., increase positive emotions) when there is frequent positive feedback. The results of this prediction indicated that CNV amplitudes were larger, and social pain scores had a significant trend to be lower (more positive) when participants were holding a soft than a hard cushion when they received the ball with high probability. The CNV amplitude is related to expectations of an imperative stimulus (Van Boxtel and Brunia 1994a, b; Ruchkin et al. 1986). Therefore, the higher CNV amplitude in the over-inclusion block of the soft cushion condition likely reflects the higher expectation that computer-generated players would throw the ball to the participant. Moreover, there was a significant trend for subjective ratings of social pain to be lower when participants held a soft than a hard cushion, which was opposite to the results of our previous study (Ikeda and Takeda 2019) in which social pain was higher in the soft cushion condition under frequent negative but not positive feedback. These results are not conclusive because subjective rating results were only marginally significant. Nevertheless, this study’s results supported the hypothesis that holding a soft object does not directly influence social pain in the Cyberball task, but it could influence social pain through the mediation of expectations.

This study did not show an effect of the cushion on the P3, which is consistent with our previous study (Ikeda and Takeda 2019). As mentioned in the Introduction, P3 develops after ball movements; therefore, it might reflect the processes of evaluating the subjective probability of ball possession, whereas the CNV might reflect preparatory processes related to expectations. Therefore, the results of this study together with our previous findings indicated that holding soft objects influenced the preparation but not the evaluation of being included in the Cyberball task by others. It is also well known that the P3 is sensitive to the arousal level (Olofsson et al. 2008). Therefore, the finding of no cushion effect on the P3 supported the notion that arousal levels of participants holding a soft cushion was not different from that of participants holding a hard cushion, which excluded the possibility that CNV results were caused by the difference in the arousal level. In this study, the P3 amplitude was larger in micro-rejection trials than in inclusion trials irrespective of the cushion condition. Participants doing the Cyberball task in this study conducted an over-inclusion block in which they had many experiences of being included by others. Therefore, unlike in our previous study, participants in this study might have estimated micro-rejection trials as rare events, which resulted in the larger P3.

It should be noted that the Cyberball task used in the present study is different from a typical paradigm used in CNV studies in terms of response preparation; i.e., this Cyberball task did not require quick motor responses, unlike a typical CNV paradigm. Therefore, the negative-going slow potential observed in the present study might include the stimulus-preceding negativity (SPN), which also reflects the expectation of forthcoming feedback (Brunia and Damen 1988; Chwilla and Brunia 1991). The dissociation between the CNV and SPN is beyond the scope of this study. Therefore, we have continued to use the term, CNV, similar to our previous study (Ikeda and Takeda 2019).

Our previous study (Ikeda and Takeda 2019) that examined a fair-play block and an exclusion block (i.e., the ball was tossed between two other players on most trials) reported that holding a soft cushion increased CNV amplitude irrespective of block type. On the other hand, the present study revealed that holding a soft cushion increased CNV amplitudes in the over-inclusion block but not in the fair-play block. A possible explanation is that a floor effect occurred in the fair-play block; that is, the CNVs elicited in the fair-play block may have been too small for the effect of cushion type to be detected. Further studies are needed to clarify this issue.

We mention here the relationship between CNV amplitudes and subjective ratings of social pain. Our previous study, which included fair-play and exclusion blocks, reported that holding a soft cushion increased both CNV amplitudes and subjective ratings of social pain (Ikeda and Takeda 2019). By contrast, this study, which included fair-play and over-inclusion blocks, found that holding a soft cushion increased CNV amplitudes but decreased subjective ratings of social pain at a marginally significant level. That is, holding a soft cushion may consistently increase the expectation of social inclusion irrespective of context (the probability of inclusion), but its effects on emotions depend on context.

The findings of this study are limited because it did not use a typical Cyberball task. That is, many repeated trials were needed in each condition to compute the ERPs; we designed this study to include two fair-play and two over-inclusion blocks in each cushion condition. Moreover, we used a quasi-random order such that the first block in each cushion condition was always a fair-play block because the fair-play block provided a baseline experience of the Cyberball task that probably resulted in an unbiased mental set for the task, which is not the typical procedure in Cyberball tasks. However, the similar procedure has often been used in previous studies, and it is useful for examining physiological measures. For example, each participant in a functional magnetic resonance image study by Kawamoto et al. (2012

2012) conducted four fair-play, four exclusion, and four over-inclusion blocks. It has also been reported that the order of fair-play and over-inclusion blocks in a Cyberball task does not affect subjective ratings (Niedeggen et al. 2014). However, repeated fair-play and over-inclusion experiences and the quasi-random order manipulation might have affected this study’s results. Therefore, careful validation is needed when discussing differences between the fair-play and over-inclusion blocks. Nevertheless, this study’s primary purpose was to investigate the effects of holding a soft cushion, and results of the CNV condition showed no significant order effects (first holding a soft cushion vs. first holding a hard cushion). Therefore, the results concerning the cushion effect are considered reliable.

In summary, this study demonstrated that holding a soft object can increase CNV amplitude and potentially decrease social pain in the Cyberball task if the participants are sufficiently included. The present results, together with the findings of our previous study (Ikeda and Takeda 2019), support the notion that holding soft objects does not directly influence emotions in the Cyberball task, but rather can influence emotions through the mediation of expectations.

## Data Availability

All relevant data are within the paper and its “Supporting Information.”
